# Silicon based mid-IR super absorber using hyperbolic metamaterial

**DOI:** 10.1038/s41598-017-18737-5

**Published:** 2018-02-01

**Authors:** Mai Desouky, Ahmed M. Mahmoud, Mohamed A. Swillam

**Affiliations:** 1 Department of Physics, The American University in Cairo, Cairo, 11835 Egypt; 2 Electronics and Communications Engineering Department, The American University in Cairo, Cairo, 11835 Egypt

## Abstract

Perfect absorbers are indispensable components for energy harvesting applications. While many absorbers have been proposed, they encounter inevitable drawbacks including bulkiness or instability over time. The urge for CMOS compatible absorber that can be integrated for on chip applications requires further investigation. We theoretically demonstrate Silicon (Si) based mid IR super absorber with absorption (*A*) reaching 0.948. Our structure is composed of multilayered N-doped Si/ Si hyperbolic metamaterial (HMM) integrated with sub-hole Si grating. Our proposed structure has tunable absorption peak that can be tuned from 4.5 µm to 11 µm through changing the grating parameters. We also propose two grating designs integrated with N-doped Si/ Si HMM that can achieve wide band absorption. The first grating design is based on Si grating incorporating different holes’ height with (*A*) varying between 0.83 and 0.97 for wavelength from 5 µm to 7 µm. The second grating design is based on Si grating with variable holes’ diameter; the latter shows broad band absorption with the maximum (*A*) reaching 0.97. We also show that our structure is omnidirectional. We propose an all Si based absorber which demonstrates a good candidate for thermal harvesting application.

## Introduction

Energy harvesting and handling is an important aspect required for several applications ranging from Microwave range ex: stealth application^1^ to near IR ex: thermal photovoltaic^2^. Heat losses associated with electronic and/or electro-optical devices is a major drawback that affects the performance of electronic/photonic circuits. In order to control the thermal losses in such devices, CMOS compatible electromagnetic wave absorber is an ultimate requirement. Electromagnetic wave absorbers are classified into; i) single energy absorbers and ii) broad band absorbers (BBA). Single absorbers are single frequency absorbers, at certain wave length, unity absorption could be realized when impedance is perfectly matched with the surrounding medium^3,4^. On the other hand, BBA can be realized through multiple ways in which among  is using multiple resonators so that several absorption peaks can be coupled^3,5^. Unfortunately, broad band absorbers based on coupled resonators results in increase in total thickness of the designed absorber^3,5^. Other structures have been proposed which are relieved from the strict impedance matching requirement and demonstrated wide bandwidth operation. Among these absorbers are; porous Si absorber^6^, Si Nano wires^7^ and metallic plasmonic particles^8,9^. Even though these structures have shown high absorption values they encounter major drawbacks including bulkiness^6,7^ as in the case of Si nano wires, others may suffer instability over time as in the case of synthesized plasmonic metal particles^9^.

Metamaterials are promising candidate for super absorbers (near unity absorbers) of smaller thickness. Metamaterials have gained increased attention over the past years after revisiting the theoretical proposals of Veselago^10^ on negative index medium. Now, metamaterials have extended their avenues to include applications in super focusing^11,12^, sub-wavelength imaging^13^ and super absorption^14,15^. Several studies showed that metamaterials could be used to achieve single and broadband absorption using specifically engineered metal/dielectric stacks, namely Hyperbolic Metamaterial (HMM**)**. HMM is characterized by its anisotropic permittivity tensor components, where it behaves as a metal in one dimension (ε < 0) and as a dielectric (ε > 0) in the other dimension^16^. This hyperboloid iso-frequency surface allows the HMM to exhibit unique optical and physical properties that cannot be found in any natural occurring materials. Among these properties is coupling of high propagation wave vectors -which are evanescent in vacuum- into propagating modes in the HMM. In addition, HMMs have an open/unbound hyperboloid dispersion which inherit them the property of having large photonic density of states^16,17^. These aforementioned properties have made the HMMs widely explored for absorption application. A previous study demonstarted a HMM with reduced reflection upon using ITO nano particles to scatter the incident field inside the HMM^18^. Another theoretical study showed that surface roughening within the HMM layers’ result in BBA^19^. Many studies have reported BBA using trapezoidal-shaped HMMs^14,20,21^. The tapered hyperbolic layers have slow light modes that enhance light confinement to the hyperbolic wave-guided tapers. Nevertheless, the control over fabrication of such structure is still a major challenge. Another design has been reported by K.Sreekanth *et al*.^22^ showing that introducing a hole grating on HMM can excite lossy modes of Bulk Plasmon Polaritons (BPPs) which can lead to broadband absorption. By designing a diffraction grating with proper dimension, the wave vectors of incident light can be coupled with the HMM wave vectors leading to near unity absorption^23^. On the other hand, CMOS compatible BBA absorber that is essential for harvesting thermal energy for on-chip applications is still of extreme importance nowadays. K.Gorgulu *et al*. have proposed a broadband mid IR Si based absorber in which BBA originated from free carrier absorption and plasmonic resonances^24^. The structure, however, included an electrically large p-doped substrate in which the broadband behavior can be attributed.

In this work, we introduce single and broad band absorber in the mid IR wave length range using a fully Si based HMM. Our structure is composed of sub-hole Si grating on top of N-doped Si/Si HMM. Our work is classified into three sections; first we show that high absorption can be achieved by introducing sub-hole Si grating on N-doped Si/Si HMM reaching a maximum absolute absorption (*A*) of 0.948. Secondly, we show that the absorption peak can be widely and easily tuned by changing the dimensions of the grating (hole’s diameter and hole’s height) across the mid-IR range. Thirdly, we propose two unit cells of sub-hole Si grating integrated on N-doped Si/Si HMM to achieve broad band absorption reaching a maximum (*A*) of 0.97. Our structure also shows minimal angle dependence, another very important requirement for efficient energy harvesting.

## Results

### Design and theoretical simulations

**Figure 1:**
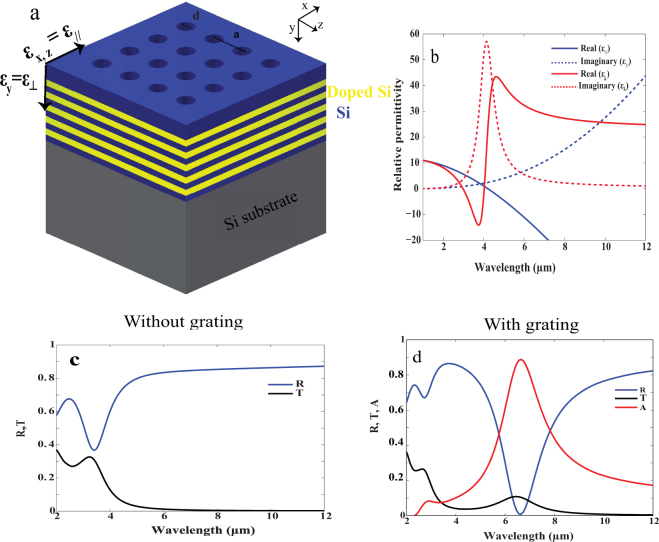
A schematic for our HMM. **(a)** 10 alternating layers of N-doped Si (yellow) and Si (blue), integrated with Si grating containing periodic air holes (blue). The period (**a)** is 500 nm, the height of hole is given by (**h)** while the diameter is given by **(d)**. The effective permittivities are defined such that *ε*_*||*_ is along the x and z-axis while *ε*_⊥_ is along the y axis. **(b)** Parallel (blue) and perpendicular (red) effective permittivity of N-doped Si/Si HMM using equations 1 and 2. (**c,d**) R (blue), T (black) and *A* (red) for bulk effective medium of N-doped Si/Si HMM, (**c)** without Si grating, and (**d)** with sub-hole Si grating.

The proposed structure is composed of 10 alternating layers of N-doped silicon acting as metal and silicon acting as dielectric. Negative perpendicular and positive parallel permittivity for HMM are defined as shown in Fig. 1a. Each layer is of thickness 50 nm and the whole structure is supported on Si substrate. A Si grating of etched holes with height **h** and diameter **d** was introduced on top of the HMM with a period of 500 nm. We start by using the effective medium theory (EMT**)** to predict the dispersion behavior of N-doped Si/Si HMM^25^. Effective permittivity in the perpendicular and the parallel directions is given by *ε*_⊥_ = *ε*_*y*_ < 0 and *ε*_||_ = *ε*_*x,z*_ < 0 respectively.

1 $${\varepsilon }_{\perp }=\frac{{\varepsilon }_{m}{\varepsilon }_{d}}{{f}_{1}{\varepsilon }_{m}+{f}_{2}{\varepsilon }_{d}}$$

2 $${\varepsilon }_{\parallel }={f}_{1}{\varepsilon }_{m}+{f}_{2}{\varepsilon }_{d}$$

where *ε*_*m*_ and *ε*_*d*_ are the permittivities of N-doped Si and intrinsic Si respectively*. f*_1_ and *f*_2_ are the filling ratios of N-doped silicon and silicon respectively. Figure 1b shows the hyperbolic dispersion of N-doped Si/Si HMM with type І hyperbolic dispersion (where *ε*_⊥_ < 0 and *ε*_*||*_ > 0) for wavelength range 2.9 μm < λ < 4.1 μm. Type Π hyperbolic dispersion is shown (where *ε*_⊥_ > 0 and *ε*_*||*_ < 0) for wavelength range 4.2 μm < λ < 12 μm. Furthermore, as a step towards simplifying and optimizing our design, we again apply EMT to study the behavior of the effective sub-hole Si grating (hole dimensions: d = 100 nm and h = 300 nm) on effective bulk N-doped Si/Si HMM as follows^25^. The effective permittivity for Si with air holes can be expressed by parallel and perpendicular permittivity *ε*_*||grating*_ and *ε*_⊥*grating*_ respectively^16^.

3 $${\varepsilon }_{\parallel grating}=\frac{(1+\rho ){\varepsilon }_{air}{\varepsilon }_{si}+(1\,-\,\rho ){\varepsilon }_{si}^{2}}{(1+\rho ){\varepsilon }_{si}+(1\,-\,\rho )\varepsilon air}$$

4 $${\varepsilon }_{\perp grating}=\rho {\varepsilon }_{air}+(1-\rho ){\varepsilon }_{si}$$

5 $$\rho =\frac{Are{a}_{hole}}{Are{a}_{unitcell}}$$

where *ε*_*air*_ and *ε*_*si*_ are permittivities for air and Si respectively, *ρ* is the filling ratio, Area_hole_ and Area_unitcell_ are the surface area of the hole and the unit cell respectively. Figure 1c shows simulation results for bulk effective medium of N-doped Si/Si HMM without Si grating (simulated using equations 1 and 2). The absolute reflection (R) is about 0.9 while the absolute transmission (T) is nearly zero. This occurs due to the fact that HMM strongly behaves as metal in the parallel direction where it becomes highly reflective beyond the Brewster angle^26^. In order to calculate the (*A*) for the following sections, we used the following formula; *A* (ω) = 1 − (R (ω) + T (ω)). Figure 1d shows R and T for bulk effective medium of N-doped Si/Si HMM loaded with the effective sub-hole Si grating (simulated using equations 3 and 4). It shows that R and T drops significantly while *(A)* of value 0.9 is observed around wavelength of 6.8 μm. This can be explained as follows: In absence of the grating, the coupling of the incident plane wave into the high propagation K modes of the HMM is impossible. The diffraction grating can however be used as an efficient mean to couple the incident light with the high propagation K modes when high order diffraction grating “of order” greater than that of light is applied on HMM. Enhanced wave vector of the grating allows for additional momentum to the incident light where coupling condition can then be satisfied^27^. Once coupling is fulfilled, noticeable resonance occurs leading to high absorption mechanism^23^. In order to confirm that this absorption comes from the grating coupled to HMM, Fig. S1a shows zero absorption for both cases of; Si grating alone and Si grating on perfect reflector, Fig. S2b (*see supplementary information*).

### Single band absorption

**Figure 2:**
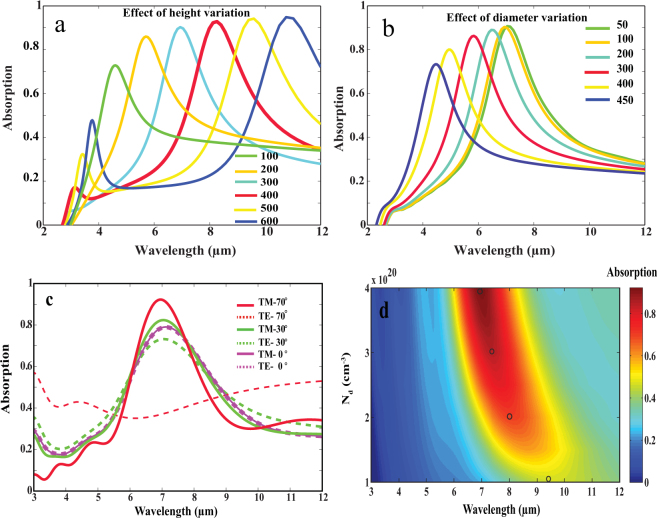
Absorption spectrum for sub-hole Si grating on N-doped Si/Si HMM. **(a)** d is fixed at 100 nm while h varies from 100 to 600 nm. **(b)** d varies from 50 to 450 nm while h is fixed at 300 nm. Oblique incidence of angle 70 degrees was used for both cases (**a**) and (**b**). **(c)** TM and TE modes at three angles of incidence (0, 30 and 70 degrees) were studied for grating of dimensions d = 100 nm and h = 300 nm. **(d)** Color map showing absorption at different doping concentrations N_d_.

Now that we verified the physical mechanism of absorption in our structure, we proceed to study in more details the effect of different geometrical aspects of the structure upon its behavior and provide a clear pathway for designing efficient CMOS compatible absorber. First, we study the effect of varying h while keeping d fixed at 100 nm. Figure 2a shows that increasing h from 100 nm to 600 nm results in red shift of the absorption peak from wavelength 4.5 μm to 11 μm. Absorption varies between 0.72 at wavelength of 4.55 μm to 0.948 at wavelength of 10.75 μm. Increasing h results in larger volume between two corresponding adjacent holes within which the energy gets confined within. The larger volume (for larger heights) will lead to lowering the confined energy and thus the absorption peak will be red shifted.

It could be also seen that the bandwidth of the absorption peak increases slightly at larger wavelengths. This has been demonstrated by K.Sreekanth *et al*.^22^ that the bandwidth of the absorption peak is affected by the contrast in permittivity between the grating and the HMM. The contrast between silicon grating and effective parallel permittivity of the HMM increases at larger wavelengths. Figure 1b shows that the magnitude of the parallel permittivity increases for larger wavelength with respect to the dielectric permittivity of intrinsic Si (11.7); this explains the slight increase in bandwidth. We can also see very good agreement between (*A*) obtained for height 300 nm at λ = 7 μm, shown in Fig. 2a and the same structure studied and demonstrated by EMT in Fig. 1d. This verifies that the EMT works as a good approximation to predict the behavior of our proposed structure. The same effect of tuning the absorption peak can be realized by changing d from 50 nm to 450 nm while h is fixed at 300 nm. Figure 2b shows that increasing the diameter from 50 nm to 450 nm results in blue shift of the absorption peak from wavelength of 7 μm to wavelength of 4.5 μm. Again, this could be understood in terms of volume where the energy gets confined within. Increasing the hole diameter results in smaller volume between two adjacent hole pillars, therefore, energy confined in smaller volume becomes higher, and thus the absorption peak will be blue shifted. Another key feature in this design is its relative independence over the angle of incidence, a very useful feature for an absorber to acquire. Fig. S2 shows that R drops for all angles from 0 to 70 degrees. The maximum reflection is about 0.17 for normal incidence and near 0 for oblique incidence of angle 70 degrees. In addition, since the grating is periodic in x and z direction, high absorption values can be addressed for TE polarized light as well. Figure 2c shows that high absorption values of nearly 0.79 are fulfilled for TE mode which is exactly equal to the TM mode absorption value in case of normal incidence. Increasing the angle of incidence to 30 degrees for TE mode results in slight decrease in absorption compared to the TM mode. Further increase in angle of incidence to 70 degrees reduces the absorption of the TE mode. Generally, 2D grating allows for another degree of freedom while designing polarization less-dependent absorbers. We also studied the effect of changing doping concentration (N_d_) on the absorption peak for grating of dimensions (d = 100 nm and h = 300 nm). Figure 2d shows that for lower doping concentrations beyond our previously studied doping concentration of (N_d_ = 4 × 10^20^ cm^−3^), there is a red shift in the absorption band and reduction in absorption value. The black dots in Fig. 2d corresponds to the maximum absorption at four different N_d_ which are; 4 × 10^20^, 3 × 10^20^, 2 × 10^20^, 1 × 10^20^ cm^−3^ that results in maximum (*A*) of 0.92, 0.85, 0.7 and 0.45 respectively. This can be illustrated as follows: decreasing the doping concentration shifts the plasma wave length for doped Si to larger wave length. As a consequence, the hyperbolic behavior “which aids the absorption mechanism” will only be supported at longer wave length as well. Fig. S3 shows the dispersion behavior for the least studied N_d_ of 1 × 10^20^ cm^−3^; it is obvious that type П hyperbolic dispersion is realized within wave length range of 8.2 μm < λ < 12 μm (where absorption band is expected to occur). However, in Fig. S3 the perpendicular imaginary part of the permittivity becomes very broad which results in noticeable reduction in absorption to nearly 0.45. Thus, it is important to design the absorber so that the resonance occurs away from the hyperbolic regime as shown in the dispersion curve in Fig. 1b.

**Figure 3:**
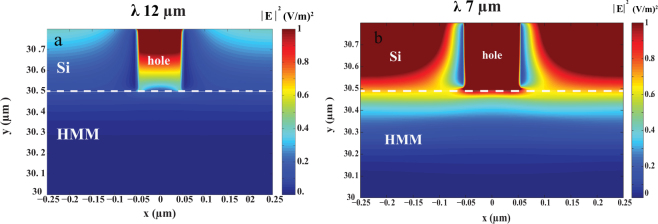
Electric field distribution |*E*|^2^ (V/m)^2^ in XY plane for sub-hole Si grating of dimensions (d = 100 nm and h = 300 nm) on N-doped Si/Si HMM for wavelength **(a)** 12 μm and **(b)** 7 μm.

The electric field distribution was simulated for sub-hole Si grating (d = 100 and h = 300 nm) on N-doped Si/Si HMM. Figure 3(a,b) shows the electric field distribution |*E*|^2^ for the XY plane for the sub-hole Si grating on N-doped Si/Si HMM. Figure 3a shows that there is no electric field generated at wave length of 12 μm in the grating or the HMM. This trend also holds over a wavelength range of 8.5 μm to 12 μm due to the reflective nature of the structure within this range. Figure 3b shows that electric field confinement in the grating and in the N-doped Si/Si HMM at the resonance wave length 7 μm as expected.

### Broad band absorption

**Figure 4:**
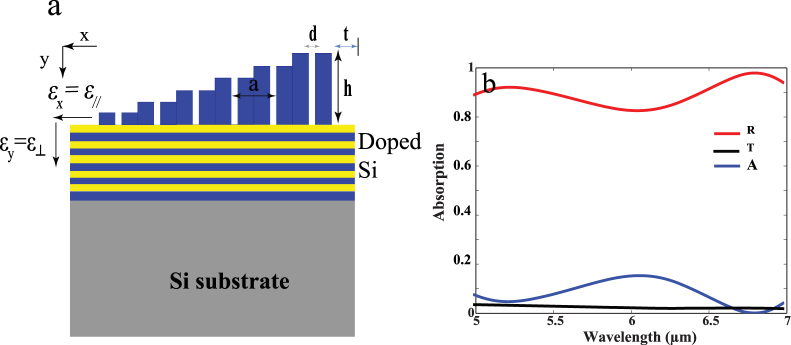
Schematic for BBA HMM. (**a**) 10 alternating layers of N-doped Si (yellow) and Si (blue) HMM with multi height sub-hole Si grating (MHSG). The period a is 500 nm, d is 50 nm, t is 300 nm and h are taken to be; 100, 200, 300, 400, 500 and 600 nm. (**b**) R, T and A for MHSG on N-doped Si /Si HM at normal incidence.

From previous work, we concluded that depending on the grating dimension absorption within certain wavelength range could be realized. In order to obtain BBA, multiple resonance mechanisms have to be supported at different wavelengths, where perfect coupling between wave vectors of incident light and that for the hyperbolic modes has to be fulfilled. In this section, a unit cell of **m**ulti **h**eight sub-hole **S**i **G**rating (**MHSG)** was proposed to realize BBA. Each unit cell is composed of six holes of different heights ranging from 100 to 600 nm on the same previously studied N-doped Si/Si HMM as shown in Fig. 4a. The diameter **d** and period **a** are fixed at 50 and 500 nm respectively. The spacing between each unit cell and the other is 600 nm. Figure 4b shows R, T and A for normal TM injection on MHSG N-doped Si/Si HMM. It can be clearly seen that BBA is achieved from 5 μm to 7 μm with *(A)* varies between 0.83 and 0.97 (minimum and maximum absorption values are selected within the specified wave length range). The BBA can be achieved at oblique incidence too as indicated in Fig. S4.

**Figure 5:**
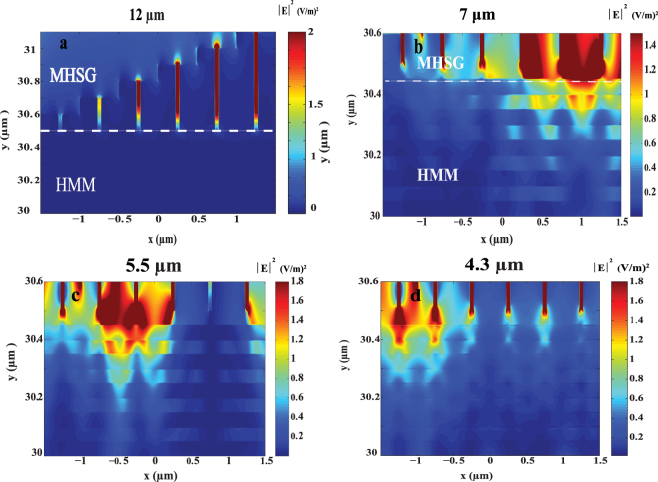
Electric field distribution |*E*|^2^ (V/m)^2^ for multi dimension sub-hole Si grating (MHSG) on N-doped Si/Si HMM at different wave lengths; (**a**) 12 μm, (**b**) 7 μm, (**c**) 5.5 μm and (**d**) 4.3 μm.

Electric field distribution was simulated for the MHSG N-doped Si/Si HMM structure. Figure 5 shows the electric field distribution |*E*|^2^ at four different spectral regions. Figure 5a shows that there is no electric field observed at λ of 12 µm since the structure is reflective at λ 8.5 μm. At λ 7 μm, high field confinement is observed in the N-doped Si/Si HMM from sub-holes of h 500 and 600 nm, Fig. 5b. Moving to lower wavelength range of 5–6 μm, the mode at the 500 and 600 nm holes start to vanish whilst it appears at the middle sub-holes 200, 300 and 400 nm, Fig. 5c. Moving to lower wave length range 4–5 μm, the mode is realized from the smaller sub-holes 100 and 200 nm, Fig. 5d. Below λ of 4 μm, the structure has high T which means it will not serve as good absorber. Generally, photon coupling from air to high K medium can be achieved by using grating coupling network^27^. The quasi periodic designed grating generates quasi periodic guided modes. These modes become resonant at multiple wavelengths when different holes’ heights are introduced. These resonating modes cause strong confinement in the HMM and the grating which results in broadband absorption. Worth mentioning here the nature of hyperbolic dispersion of HMMs, the hyperboloid iso-frequency surface is an open/unbounded space which can provide large photonic density of states. The existence of available empty states enhances the incident light coupling mechanism to the doped Si /Si layers surface plasmons and aids the generation of the high lossy guided modes that causes this BBA.

**Figure 6:**
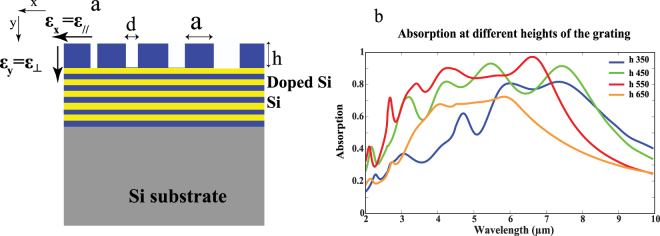
Schematic for BBA HMM. **(a)** 10 alternating layers of N-doped Si /Si with multi diameters sub-hole Si grating (MDSG). Four holes of d 100, 200, 300 and 400 nm were chosen with a of 400 nm. **(b)** Absorption at different grating heights h for MDSG on HMM: 350, 450, 550 and 650 nm.

In this section, we study the effect of BBA by proposing a grating design of multiple diameters sub-hole Si grating (**MDSG**) on N-doped Si /Si HMM. The HMM is typically as previously demonstrated in Figs 1a and 4a, the Si grating in this case however include four holes of diameters vary between 100 to 400 nm (Fig. 6a). The height **h** of the grating is fixed at 550 nm. **a** is defined as the distance between two holes and is chosen to be 400 nm. Fig. S5 shows that BBA could be realized from wavelength 4.5 μm to 7 μm where (*A*) varies between 0.84–0.972.

Figure 6b indicates the shifts in absorption band for different grating heights for the same studied MDSG. For grating of height 650 nm, the BBA is realized within the wave length range of (3.7 μm to 6 μm) while the (*A*) is nearly (0.6 to 0.71). For grating of height 550 nm, the BBA is realized within the wave length range of (2.9 μm to 7.4 μm). Reducing the grating height to 450 nm shifts the BBA to 8.3 μm while the *(A)* is slightly decreased. Further reduction in grating height to 350 nm shifts the BBA to nearly (5.5 μm to 8.3 μm) while (*A*) is reduced to nearly (0.67 to 0.8). It is obvious that, there is a slight red shift in the BBA when the height of the grating decreases from 650 to 350 nm and this contradicts our previous analysis. We previously explained that for periodic grating of fixed height, decreasing the height results in blue shift in the absorption peak (Fig. 2a) since decreasing the volume of confinement increases the confined energy and thus results in a blue shift. However, in the case of multi diameter Si grating structure, we acknowledge the fact that this system is a quasi-periodic system and its physics is much more complex than the previous single dimension hole grating. In such complex design, we should consider the interaction or interference effect of each sub-hole with one another.

## Discussion

In this theoretical study, we were able to demonstrate a mid-IR Si based super absorber of total thickness not exceeding 1 microns using HMM integrated with sub-hole Si grating. Single band absorption was achievable with (*A*) reaching 0.948. We were able to tune the absorption peak over the mid- IR range from 4.5 to 11 µm by either changing the grating hole’s height or by changing the hole’s diameter. We were able to confirm that it is an omnidirectional and less- polarization dependent absorber. The first proposed design has profound application in bio and chemical sensing mechanisms based upon tuning the single absorption peak of predesigned grating. We were also able to show that BBA can be achieved by using an all Si based structure. We proposed two grating designs, namely: Si grating with different holes’ height and Si grating with different holes’ diameter, both were integrated with the N-doped Si/Si HMM. Both designs have acquired BBA with maximum (*A*) reaching 0.97. In order to maximize the band width of the broad band absorption, further studies will be required to understand the interaction between sub-holes with one another. In addition, in order to confirm that these resonating modes are due to the fact of excitation of BPPs in HMM, a study on the electron distribution complex profile in HMM is a must. Precise identification of the bulk plasmons location based upon studying the electron distribution allows us to have dispersion relation of bulk plasmons in doped semiconductor/semiconductor HMM. This can be done as a whole study on bulk plasmons in complex structures in a future work. It should be accounted also that the effective medium approximation does not take into consideration the interaction among sub-wave length structures within the single unit cell, it is an approximation for a whole sub-wave length periodic system. This is beyond the scope of this manuscript and can be again studied in further details in a future work. However, our proposed broad band absorbers are suitable candidate for thermal harvesting application in the mid IR range. An additional advantage for our structure is that it is an all Si based absorber which consequently indicates the feasibility of being fabricated by standard Si fabrication techniques. For Metamaterial fabrication, standard chemical vapor deposition can be applied for Si layers deposition while ion beam irradiation can be used to dope the Si layers. For patterning both the periodic grating or the multiple diameters’ hole grating, photolithography and deep reactive ion etching can be used. For grating of different hole heights’, Nano imprint lithography can be used to pattern a stair case grating followed by photolithography and deep reactive ion etching. This absorber opens avenues for CMOS compatible energy harvesters for on chip purposes.

## Methods

Finite difference time domain (*Lumerical*) is used for simulating a TM polarized Plane wave incident from the top of the proposed structure. Perfect matched layer (PML) is defined along the y directions whereas Bloch Boundary conditions are defined along the x and z directions.

The permittivity of N-doped Si ε_doped_ at certain dopant concentration **N**_**d**_ is calculated using Drude model as follows:

6 $${\varepsilon }_{doped}={\varepsilon }_{\infty }-\frac{{\omega }_{p}^{2}}{1+i{\omega }^{2}{\rm{\Gamma }}}$$

7 $${\omega }_{p}^{2}=\frac{{N}_{d}\,.\,{q}^{2}}{{\varepsilon }_{0}{m}^{\ast }}$$

where ω_p_ is the plasma frequency, ɛ_∞_ is the static frequency, Γ is the damping term, m^*^ is the effective mass, q is the electronic charge. N_d_ was taken to be 4 × 10^20^ cm^−3^ which yields plasma wavelength of 2.9 μm.

### Data and materials availability

All data needed to evaluate the conclusions in the paper are present in the paper and/or the Supplementary Materials. Additional data related to this paper may be requested from the authors.

## Electronic supplementary material

Supplementary Information
