# Supplementary material for: Silicon based mid-IR super absorber using hyperbolic metamaterial
Source: Sci Rep. 2018 Feb 1;8:2036. doi: 10.1038/s41598-017-18737-5 (PMC5794863; doi:10.1038/s41598-017-18737-5)
Supplement: Supplementary file 1 — Supplementary Information [file 41598_2017_18737_MOESM1_ESM.pdf]

Supplementary Information:

Silicon based mid-IR super absorber using hyperbolic metamaterial

Mai Desouky [1], Ahmed M. Mahmoud[1, 2], Mohamed A. Swillam*[1],

[1]Department of Physics, The American University in Cairo, Cairo, 11835, Egypt

[2]Electronics and Communications Engineering Department, The American University in Cairo, Cairo, 11835, Egypt

*Corresponding author: m.swillam@aucegypt.edu

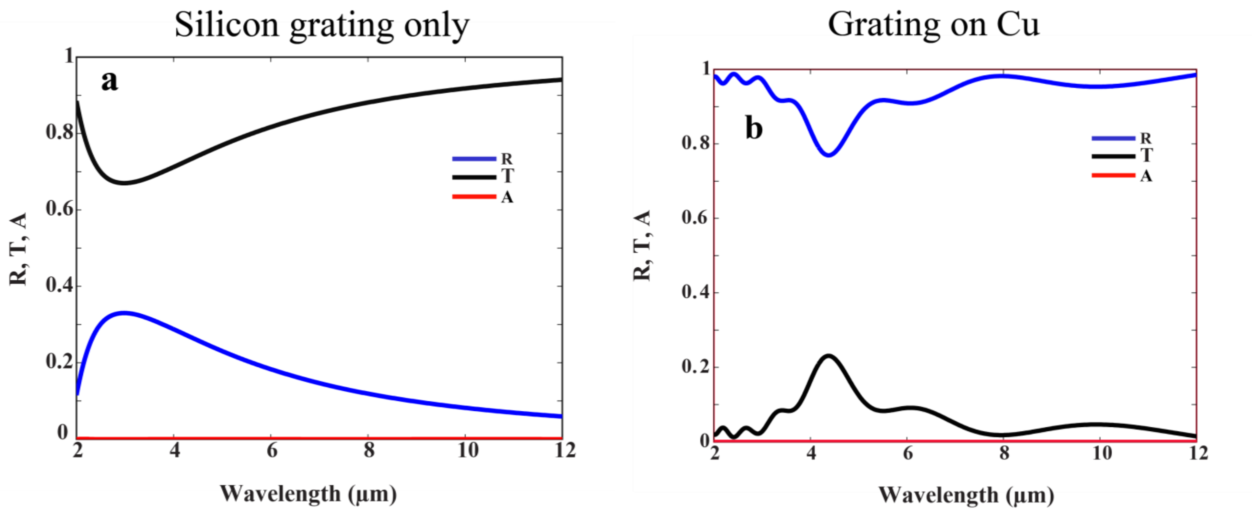

Figure S1: Reflection (R), Transmission (T) and absorption (A) spectrum. for a) Silicon hole grating only and b) Silicon grating on perfect reflector.

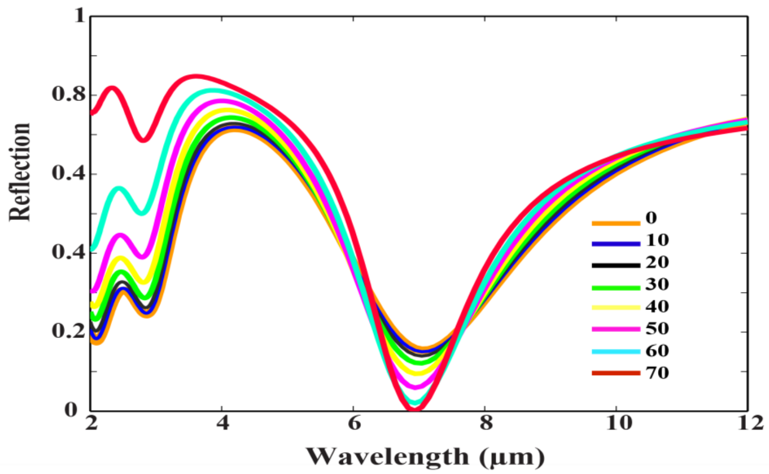

Figure S2. Reflection spectrum for uniform hole grating on HMM. Different angles of incidence from 0 to 70 degrees

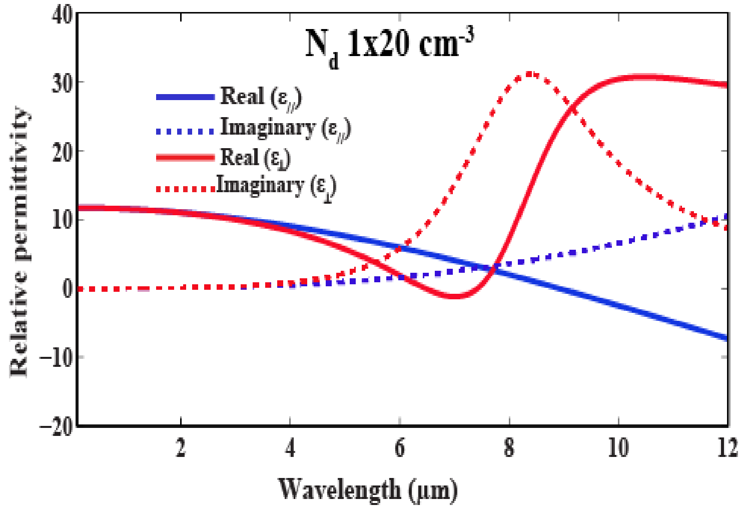

Figure S3. Dispersion relation for doped Si/Si HMM. N_d of $1x10^{20}$ cm^{-3}.

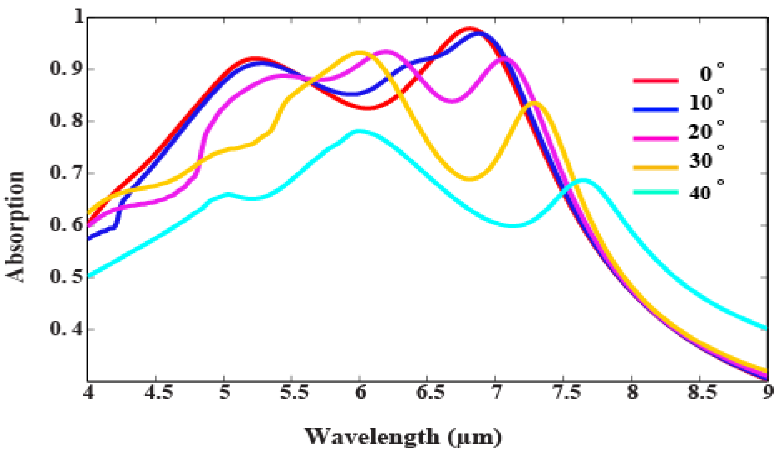

Figure S4. Broad band absorption for MHSG. Absorption for MHSG on N-doped Si /Si HMM at oblique incidence from 0 to 40 degrees.

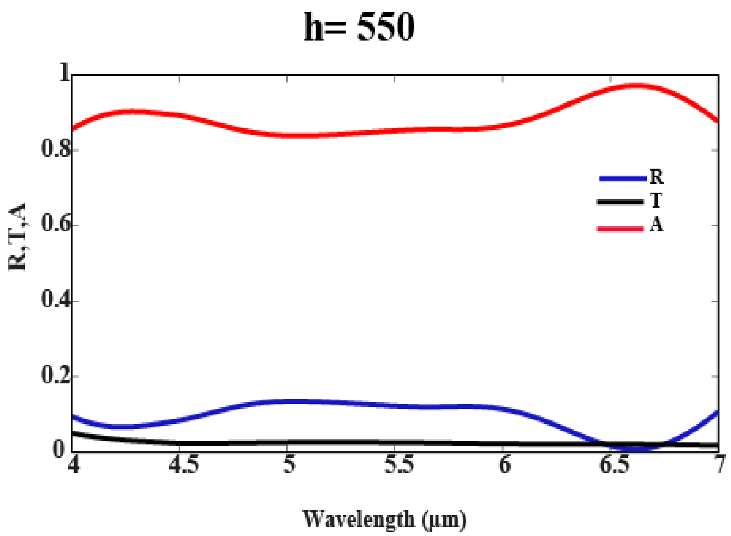

Figure S5. Broad band absorption for MDSG. R, T and A for MDSG on N-doped Si /Si HM at normal TM injection for grating of h 550 nm.
